# Passive Heating Attenuates Post-exercise Cardiac Autonomic Recovery in Healthy Young Males

**DOI:** 10.3389/fnins.2017.00727

**Published:** 2017-12-21

**Authors:** Tiago Peçanha, Cláudia L. de Moraes Forjaz, David A. Low

**Affiliations:** ^1^Exercise Hemodynamic Laboratory, School of Physical Education and Sport, University of São Paulo, São Paulo, Brazil; ^2^Research Institute for Sport and Exercise Sciences, Liverpool John Moores University, Liverpool, United Kingdom

**Keywords:** thermoregulation, heat stress, heart rate recovery, heart rate variability, aerobic exercise, autonomic nervous system

## Abstract

Post-exercise heart rate (HR) recovery (HRR) presents a biphasic pattern, which is mediated by parasympathetic reactivation and sympathetic withdrawal. Several mechanisms regulate these post-exercise autonomic responses and thermoregulation has been proposed to play an important role. The aim of this study was to test the effects of heat stress on HRR and HR variability (HRV) after aerobic exercise in healthy subjects. Twelve healthy males (25 ± 1 years, 23.8 ± 0.5 kg/m^2^) performed 14 min of moderate-intensity cycling exercise (40–60% HR_reserve_) followed by 5 min of loadless active recovery in two conditions: heat stress (HS) and normothermia (NT). In HS, subjects dressed in a whole-body water-perfused tube-lined suit to increase internal temperature (T_c_) by ~1°C. In NT, subjects did not wear the suit. HR, core and skin temperatures (T_c_ and T_sk_), mean arterial pressure (MAP) skin blood flow (SKBF), and cutaneous vascular conductance (CVC) were measured throughout and analyzed during post-exercise recovery. HRR was assessed through calculations of HR decay after 60 and 300 s of recovery (HRR60s and HRR300s), and the short- and long-term time constants of HRR (T30 and HRRt). Post-exercise HRV was examined via calculations of RMSSD (root mean square of successive RR intervals) and RMS (root mean square residual of RR intervals). The HS protocol promoted significant thermal stress and hemodynamic adjustments during the recovery (HS-NT differences: T_c_ = +0.7 ± 0.3°C; T_sk_ = +3.2 ± 1.5°C; MAP = −12 ± 14 mmHg; SKBF = +90 ± 80 a.u; CVC = +1.5 ± 1.3 a.u./mmHg). HRR and post-exercise HRV were significantly delayed in HS (e.g., HRR60s = 27 ± 9 vs. 44 ± 12 bpm, *P* < 0.01; HRR300s = 39 ± 12 vs. 59 ± 16 bpm, *P* < 0.01). The effects of heat stress (e.g., the HS-NT differences) on HRR were associated with its effects on thermal and hemodynamic responses. In conclusion, heat stress delays HRR, and this effect seems to be mediated by an attenuated parasympathetic reactivation and sympathetic withdrawal after exercise. In addition, the impact of heat stress on HRR is related to the magnitude of the heat stress-induced thermal stress and hemodynamic changes.

## Introduction

Post-exercise heart rate (HR) recovery (HRR) and heart rate variability (HRV) are non-invasive tools to assess cardiac autonomic recovery after exercise (Imai et al., 1994; Peçanha et al., 2014) and can predict cardiovascular disease risk (Cole et al., 1999; Peçanha et al., 2014; Pradhapan et al., 2014). HRR presents a biphasic pattern, with a fast initial phase that is determined by parasympathetic reactivation followed by a secondary slow decay that is mediated by continued parasympathetic reactivation and sympathetic withdrawal (Perini et al., 1989; Imai et al., 1994). Several studies have investigated the mechanisms underlying these post-exercise autonomic responses (Carter et al., 1999; Peçanha et al., 2014, 2016) and exercise-induced thermoregulatory demands have been suggested to play an important role (Peçanha et al., 2014; Michael et al., 2017).

During exercise, increased muscle metabolism results in elevated body heat storage, which leads to an increase in core temperature (T_c_; Saltin and Hermansen, 1966; Crandall and González-Alonso, 2010). To prevent dramatic increases in T_c_, heat loss mechanisms, such as skin vasodilation and sweating, are activated (Journeay et al., 2006; González-Alonso, 2012). These responses are accompanied by neurally-mediated increases in heart rate (HR) and vasoconstriction of non-active vascular beds (González-Alonso, 2012; Kenney et al., 2014) to allow for the maintenance of blood pressure within appropriate limits (Kenney et al., 2014). After exercise, accumulated body heat is gradually lost, and evidence suggests that HRR tracks the reduction of T_c_ after exercise (Franklin et al., 1993; Leicht et al., 2009; Lynn et al., 2009). For example, Leicht et al. (2009) observed faster post-exercise decays of T_c_ and HR when subjects were cooled after exercise. Likewise, Lynn et al. (2009) compared the hemodynamic responses after exercise performed in different thermal conditions, and observed a delay in the recoveries of T_c_ and HR in warm compared with thermoneutral conditions. Such evidence supports the role of thermoregulation in post-exercise HR regulation, however these previous studies assessed HRR for 10–90 min after the end of exercise, but not at the onset of recovery (i.e., immediately after exercise), a period known to be critical for post-exercise cardiac autonomic regulation analysis (Peçanha et al., 2014, 2017a) and for predicting cardiovascular disease risk (Cole et al., 1999; Peçanha et al., 2014). Furthermore, in the study of Lynn et al. (2009), besides differences in T_c_ between warm and thermoneutral conditions, HR was also different during exercise, which could have affected post-exercise results. In addition, in the study of Leicht et al. (2009), although HR was paired in control and cooling sessions during the exercise, T_c_ was not different prior to the post-exercise period. An ideal approach to investigate the role of thermoregulation on HRR would be to provoke differences in T_c_ between the conditions but ensure similar HRs during the exercise. To date, such a study has not been conducted.

A potential association between thermoregulation and HRR might involve T_c_ induced changes in systemic and/or skin hemodynamics (i.e., mean arterial pressure—MAP and skin blood flow—SKBF, respectively). In this respect, a reduction in MAP during post-exercise recovery in heat stress has been associated with increased HR (i.e., reduced HRR; Franklin et al., 1993). However, at this point, little is known about the effects of different thermal conditions on the associations between HRR and thermoregulatory/hemodynamic responses. Therefore, the aim of this study was to assess the effects of heat stress on post-exercise HRR immediately after aerobic exercise and the association between heat stress-induced changes in HRR and in thermoregulatory and hemodynamic responses. Our hypotheses were that heat stress would reduce HRR and HRV, and the reductions in HRR would be associated with thermal and hemodynamic responses imposed by the heating protocol.

## Materials and methods

### Subjects

Twelve healthy young men (25 ± 1 years; 77 ± 2 kg; 1.80 ± 0.01 m; 23.8 ± 1.9 kg/m^2^) took part in the study. Participants were recreationally active, non-smokers, had no history of cardiovascular disease and were not taking any form of medication. After a detailed explanation of the experimental procedures, subjects provided their informed written consent. This study was conducted in accordance with the Declaration of Helsinki, and was approved by the Ethics Committee of the Liverpool John Moores University.

### Preliminary evaluation

Prior to the experimental sessions, subjects visited the laboratory for a set of preliminary exams. The subject's health status was investigated through a detailed interview. Readiness to exercise was assessed through the Physical Activity Readiness Questionnaire (PAR-Q; Shephard, 1988). Body weight and height were measured using a calibrated scale. Seated blood pressure was assessed by an automated sphygmomanometer (GE Pro300V2; Dinamap, Tampa, United States) positioned on the subjects' left arm. Afterwards, subjects performed a maximal exercise test on a magnetically braked cycle ergometer (Corival 400, Lode, Groningen, The Netherland) using an incremental step protocol. The test started with 5 min of warm-up at ~50% of the expected maximal workload. Workload was then increased by 30 watts every 2 min until maximal effort/volitional exhaustion was achieved. All subjects attained maximal workload within 8–12 min. During the test, ventilatory variables were continuously measured using a metabolic cart (CPX Ultima, Medical Graphics Corporation, Minnesota, United States) and HR was continuously recorded with a HR monitor (Polar RS800cx, Kempele, Finland). Peak oxygen consumption (VO_2peak_) and HR (HR_peak_) were determined by their maximal values at the end of the exercise test (average of 30 s).

### Experimental sessions

All subjects performed two experimental sessions (heat stress—HS and normothermia—NT) conducted at the same time of day, in a randomized order, and separated by 3–7 days. Temperature and humidity of the laboratory were kept constant across the sessions (temperature ≈22–23°C; humidity ≈35%). Subjects were instructed to avoid alcohol and exercise for 24 h, caffeine ingestion for 12 h, and food intake for 2 h prior to the sessions.

Upon arrival to the laboratory, subjects weighed themselves nude and collected their urine for urine osmolality assessment (U_osm_; Osmocheck pocket pal OSMO, Vitech Scientific Ltd, Horsham, United Kingdom). Subjects were only admitted to the protocol if their U_osm_ ranged from 200 to 600 mOsmol/kgH_2_O. The protocol started with a supine resting baseline assessment, and then the subjects were exposed to a passive heat stress (in the HS session) or a normothermic (in the NT session) intervention in the supine position. For HS, subjects were dressed in a water-perfused tube-lined suit (Med-Eng, Ottawa, Canada) covering the entire body, except for the head, face, hands, feet and the right forearm. This system controls skin and core temperature by changing the temperature of the water perfusing the suit. Subjects were exposed to HS by perfusing 48°C water through the suit until T_c_ had increased ≈1°C or for 60 min. Once the target T_c_ was reached, the temperature of the water perfusing the suit was reduced to ~42°C to limit any further increase in T_c_, and the subjects remained dressed in the suit for the remainder of the experimental protocol. For the NT intervention, subjects remained in the supine resting position for a similar timeframe, but without wearing the suit. After HS/NT interventions, subjects rested in the supine position for 10-min pre-exercise resting assessment and were then transferred to an upright cycle ergometer (Corival 400, Lode, Groningen, The Netherlands) to perform 14 min of exercise. The first 7 min of exercise were performed at 40% of the subject's HR reserve (EX1), whereas the last 7 min were performed at 60% of HR reserve (EX2). The choice of two exercise intensities was based on the purpose to verify the effects of HS on baroreflex sensitivity during exercise, data that has already been published (Peçanha et al., 2017b). To match exercise HR between the HS and NT sessions, an important requirement in studies comparing HRR between different conditions, the absolute workload was lower in the HS session. Immediately after exercise, subjects performed unloaded cycling (60 rpm) for 5 min. After the protocol, subjects re-weighed themselves nude and were instructed to rehydrate accordingly.

### Measurements

During the experimental sessions, T_c_ was measured every 10 s using a telemetric temperature pill (CorTemp® Wireless Ingestible Temperature Sensor, HQInc., Palmetto, United States) swallowed by the subjects at least 2 h prior to the experiments. This system has been shown to provide a valid T_c_ measurement at rest and during exercise (Byrne and Lim, 2007). Mean skin temperature (T_sk_) was measured through the weighted average of six thermocouples (Surface temperature probe, Ellab, Norwich, United Kingdom) (Taylor et al., 1989) and recorded continuously online (E-Val Pro, Ellab, Norwich, United Kingdom). HR was obtained using a 3 lead electrocardiogram (Powerlab, AD Instruments, Oxford, United Kingdom) and beat-by-beat blood pressure was measured on the middle finger of the right hand using photoplethysmography (Finometer, Finapress Medical System, Amsterdam, The Netherland). SKBF was measured via laser-Doppler flowmetry using an integrated flow probe (Periflux System 5001, Perimed, Jarfalla, Sweden) attached to the right forearm, and cutaneous vascular conductance (CVC) was calculated from the ratio of SKBF and finger MAP. HR, beat-to-beat blood pressure, T_c_ and SKBF were recorded continuously online (Powerlab, AD Instruments, Oxford, United Kingdom; 1 KHz sampling rate). Cardiovascular and thermoregulatory data obtained during baseline, pre-exercise and exercise in both sessions have already been presented in a previous publication (Peçanha et al., 2017b). Subsequently, in the present study, the analyses have focused on the post-exercise recovery period.

### Data analysis

#### Post-exercise cardiac autonomic recovery

Post-exercise RR interval (RRi) time series were transferred to Matlab software (Matlab 6.0, MathWorks®, Massachusetts, USA) and HRR and post-exercise HRV were assessed as previously reported (Peçanha et al., 2016).

#### Heart rate recovery

The mean absolute values of HR during the last 60 s of exercise and in segments of 30 s during the recovery were reported. In addition, the following indices of HRR were calculated: (a) HRR60s and (b) HRR300s, i.e., the absolute difference between HR during the last 60 s of exercise and the HR measured, respectively, at 60 and 300 s of recovery; (c) T30 index, i.e., short-term time constant of HRR, obtained from the negative reciprocal of the linear regression between HR and time in the first 30 s of recovery (Imai et al., 1994); (d) HRRt, i.e., long-term time-constant of HRR obtained after exponential fitting of HR during the entire 300 s of recovery (Perini et al., 1989). For T30 and HRRt, only the fittings with coefficients of determination (i.e., *R*^2^) ≥0.50 were accepted for analysis. The HRR60s and T30 reflect the fast initial phase of HRR, and for this reason, are recognized as markers of parasympathetic reactivation, whereas HRR300s and HRRt generally quantify the slow-phase of HRR, and for this reason are accepted as markers of both parasympathetic reactivation and sympathetic withdrawal (Peçanha et al., 2017a).

#### Post-exercise heart rate variability

Because of the non-stationary behavior of RRi during the post-exercise period, the assessment of HRV during this period was performed by the method proposed by Goldberger et al. (2006). Firstly, the RRi time series were processed by a median filter operation. Then, HRV was assessed through the calculation of RMSSD (square root of the mean of the sum of the squares of differences between adjacent normal RRi) and RMS (root mean square of the residuals of the linear regression of the RRi) indices on successive non-overlapped 30 s segments during the entire 5 min of recovery. RMSSD quantifies the beat-to-beat variations in RRi, and for this reason is considered a reliable marker of parasympathetic modulation (Goldberger et al., 2006), whereas RMS quantifies the overall variation of RRi around the trend line, and for this reason is considered a marker of both parasympathetic and sympathetic modulation (Ng et al., 2009).

#### Post-exercise baroreflex sensitivity

Spontaneous post-exercise baroreflex sensitivity was calculated using the sequence technique. Briefly, the software identified sequences of three or more consecutive beats in which systolic blood pressure and RRi changed in the same direction (at least 1 mmHg for SBP and 4 ms for RRi). In each sequence, the slope of the linear regression line between systolic blood pressure and RRi was determined (only sequences with *r*^2^ > 0.8 were used) and the mean of the slopes was determined as the mean baroreflex sensitivity (Parati et al., 1988).

### Statistical analysis

The Shapiro–Wilk test was employed to verify data distribution. Since, T30, HRRt, RMS and RMSSD did not present normal distribution, these data were log-transformed (ln) and normality was achieved. Paired *T*-Tests were employed to compare descriptive data, and post-exercise T_c_, T_sk_, MAP, SKBF, CVC, and HRR between HS and NT sessions. A two-way ANOVA (session vs. time) was employed to compare responses of HR and HRV between HS and NT sessions across the different time points. When a main effect or an interaction was significant, *post-hoc* comparisons were performed using the Newman–Keuls test. To assess the associations between the effects of heat stress on HRR and thermoregulatory variables, the differences between HS and NT for HRR60s, HRR300s and mean values of post-exercise T_c_, T_sk_, MAP, SKBF, and CVC were calculated, and the Pearson's product-moment correlation analysis was performed between these differences. Correlations were considered: trivial for *r* < 0.1; small for *r* = 0.1–0.3; moderate for *r* > 0.3–0.5; large for *r* > 0.5–0.7; very large for *r* > 0.7–0.9; and extremely large for *r* > 0.9 (Hopkins et al., 2009). A *p* ≤ 0.05 was considered statistically significant. All analyses were performed online using the software STATISTICA (v 8.0, StatSoft, Tulsa, United States). Data are presented as mean ± 1 *SD*.

## Results

### Baseline and exercise data

The subjects' baseline characteristics (weight: 77.0 ± 8.2 kg; height: 180 ± 6 cm; VO_2peak_: 47.3 ± 2.3 ml.kg^−1^.min^−1^; HR_peak_ 185 ± 2 bpm) as well as the pre-exercise and exercise thermoregulatory and hemodynamic data have already been reported in a previous publication from our group, which have assessed the effects of HS on baroreflex control of HR (Peçanha et al., 2017b). Subject's initial hydration status was similar between HS and NT sessions as demonstrated by similar U_osm_ (449 ± 60 vs. 470 ± 58 mOsmol/kgH_2_O; *P* = 0.72). Baseline resting HR was similar between the sessions (55 ± 9 vs. 56 ± 9 bpm, *P* = 0.23) and pre-exercise HR was greater in HS (79 ± 13 vs. 55 ± 8). Absolute exercise HR (EX1 = 116 ± 3 vs. 114 ± 3 bpm, *P* = 0.73 and EX2 = 143 ± 4 vs. 142 ± 3 bpm; *P* = 0.61, respectively) as well as percentage of HR relative to HR_reserve_ (EX1 = 40 ± 1 vs. 40 ± 1%, *P* = 0.49 and EX2 = 62 ± 1 vs. 62 ± 1%, *P* = 0.99, respectively) were similar between NT and HS. Exercise workload was significantly lower in HS (EX1 = 50 ± 9 vs. 114 ± 8 Watts, *P* < 0.01; EX2 = 106 ± 10 vs. 165 ± 8 Watts; *P* < 0.01).

### Post-exercise thermoregulatory and hemodynamic responses

**Figure 1:**
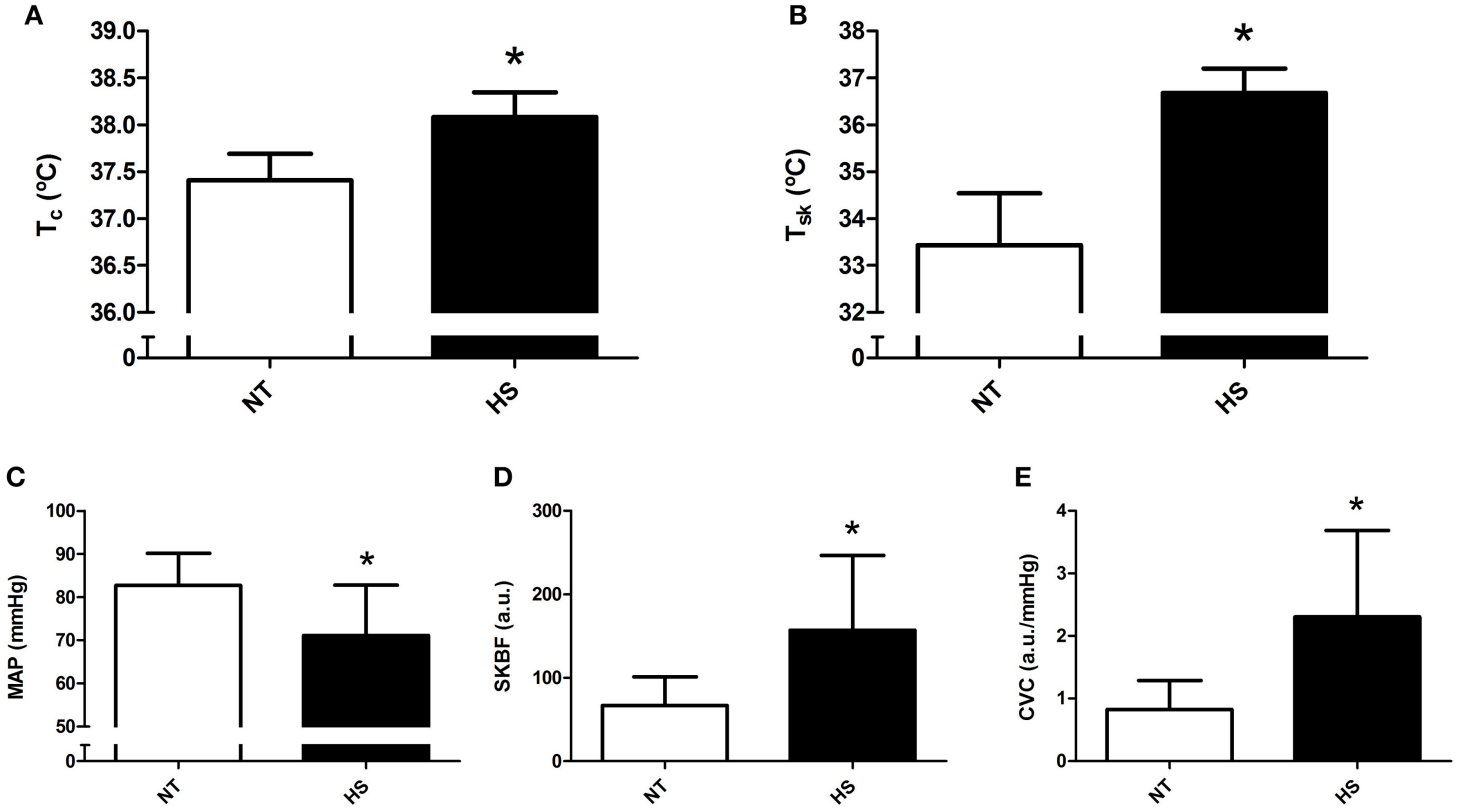
Mean post-exercise thermoregulatory and hemodynamic data obtained in the normothermic (NT) and heat stress (HS) sessions. **(A)** Core temperature (T_c_). **(B)** Skin temperature (T_sk_). **(C)** Mean arterial pressure (MAP). **(D)** Skin blood flow (SKBF). **(E)** Cutaneous vascular conductance (CVC). ^*^P ≤ 0.05 vs. NT.

Mean post-exercise T_c_, T_sk_, SKBF, and CVC were significantly higher, and MAP was significantly lower in HS than NT session (*P* ≤ 0.01 for all comparisons, Figure 1).

### Post-exercise cardiac autonomic recovery

**Figure 2:**
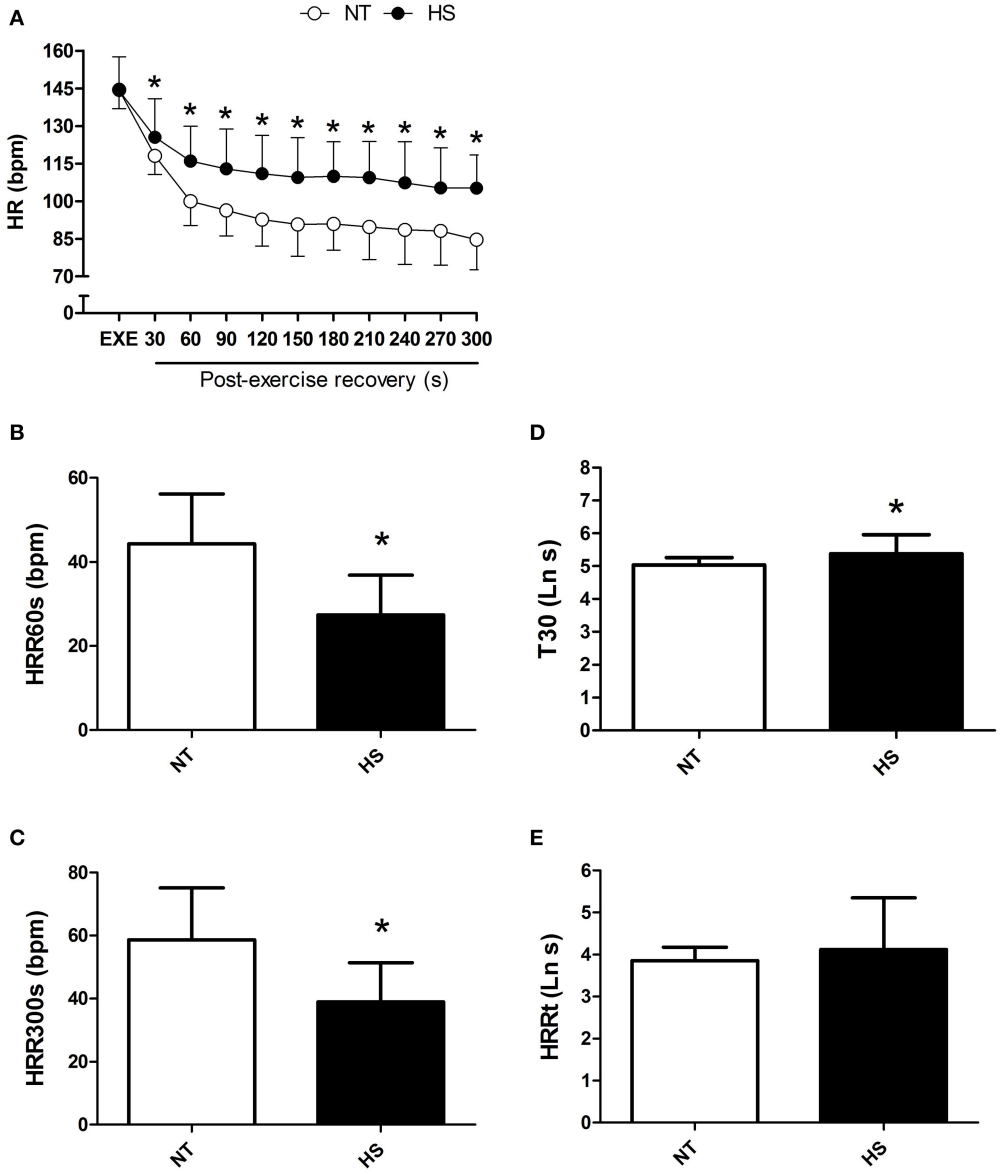
Post-exercise heart rate recovery (HRR) measured in the normothermic (NT) and heat stress (HS) sessions. **(A)** HR at the end of exercise and in 30 s segments during the recovery. **(B,C)** HRR after 60 and 300 s of recovery (HRR60s and HRR300s). **(D)** T30; short-term time-constant of HRR. **(E)** HRRt; long-term time-constant of HRR after exponential fitting. ^*^P ≤ 0.05 vs. NT. Ln, logarithm units.

HR at the end of exercise was similar between the sessions; however post-exercise HR was significantly higher in HS than NT from 30 to 300 s of recovery (*P* < 0.01 for time vs. session, Figure 2A). In addition, HRR60s and HRR300s were significantly lower in HS than NT (*P* < 0.01, Figures 2B,C). In addition, T30 index was higher in HS (*P* < 0.02, Figure 2D), with no difference between sessions for HRRt (*P* = 0.29, Figure 2E).

**Figure 3:**
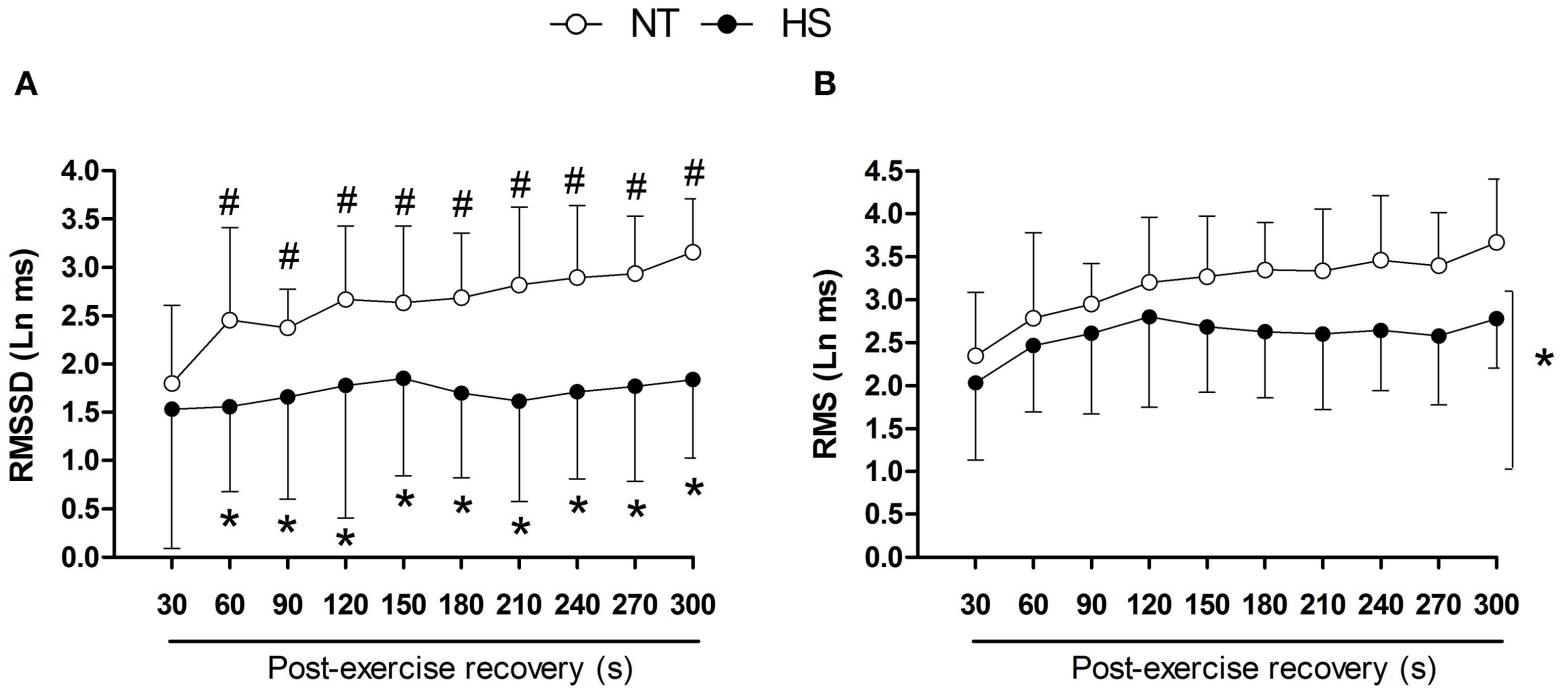
Post-exercise heart rate variability indices measured in segments of 30 s in the normothermic (NT) and heat stress (HS) sessions. **(A)** RMSSD, square root of the mean of the sum of the squares of differences between adjacent RR intervals. **(B)** RMS, root mean square of the residuals of the linear regression of the RR intervals. ^*^P ≤ 0.05 vs. NT; ^#^*P* ≤ 0.05 vs. 30 s. Ln, logarithm units.

RMSSD showed a marked increase during the recovery in NT and this increase was suppressed in HS (Figure 3A). In addition, RMSSD was lower in HS than NT from 60 to 300 s of the recovery (*P* = 0.01 for session vs. time interaction). In addition, regardless of time, RMS was lower in HS (*P* < 0.01 for session main effect, Figure 3B).

Post-exercise baroreflex sensitivity was reduced in HS compared with NT (3.0 ± 2.9 vs. 5.4 ± 2.5 ms/mmHg, *P* = 0.01).

### Associations between changes in heart rate recovery and thermoregulation/hemodynamics

**Figure 4:**
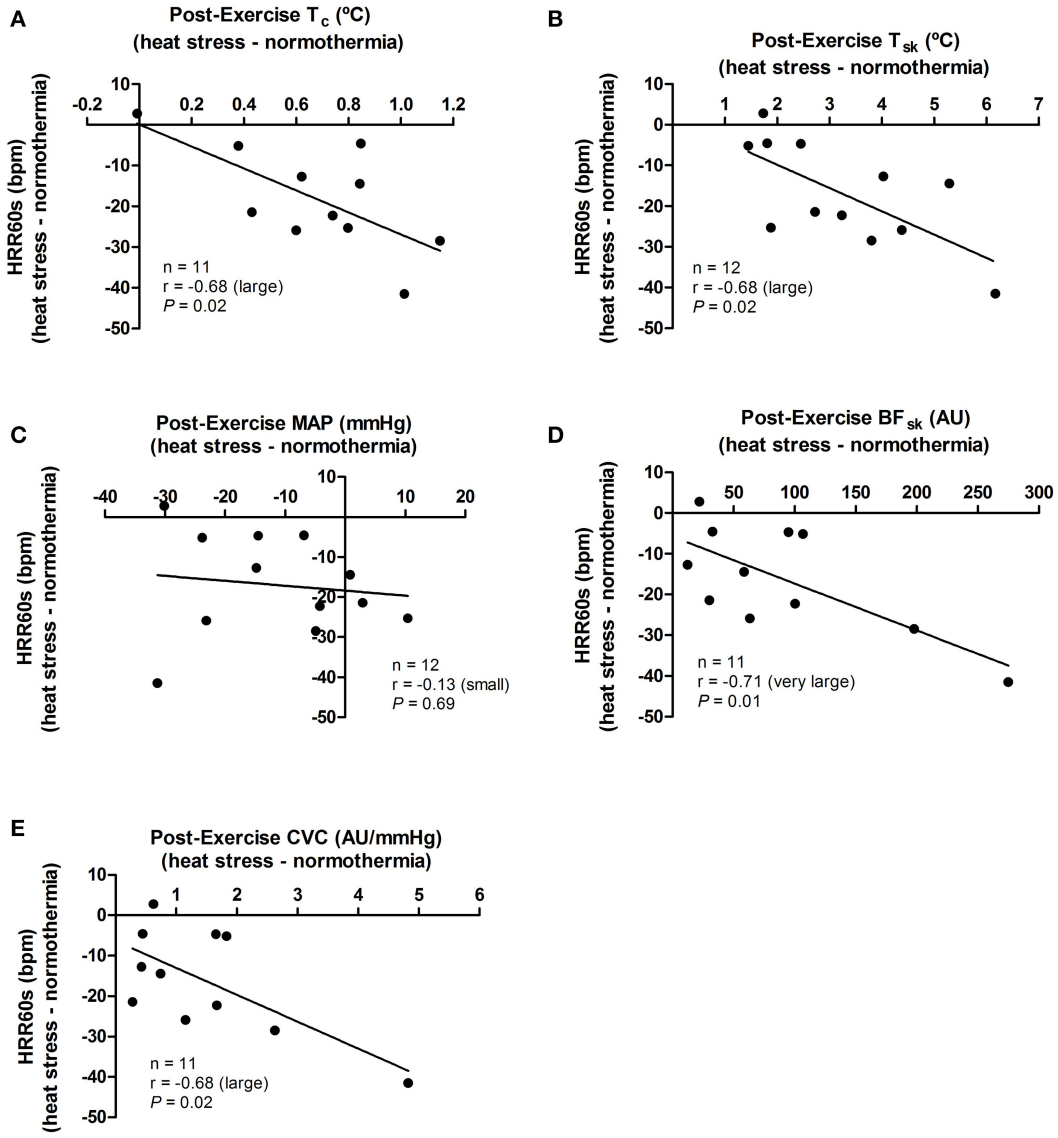
Associations between the effects of heat stress (i.e., heat stress—normothermia) on HRR60s (heart rate decay after 60 s of recovery) and on thermoregulatory and hemodynamic variables. **(A)** T_c_, core temperature; **(B)** T_sk_, skin temperature; **(C)** MAP, mean arterial pressure; **(D)** SKBF, skin blood flow; **(E)** CVC, cutaneous vascular conductance. Due to 1 missing data point of T_c_, SKBF and CVC, correlations are presented for 11 subjects. Correlations (*r*) were classified as trivial (<0.1), small (0.1–0.3), moderate (>0.3–0.5), large (>0.5–0.7), very large (>0.7–0.9), and extremely large (>0.9).

**Figure 5:**
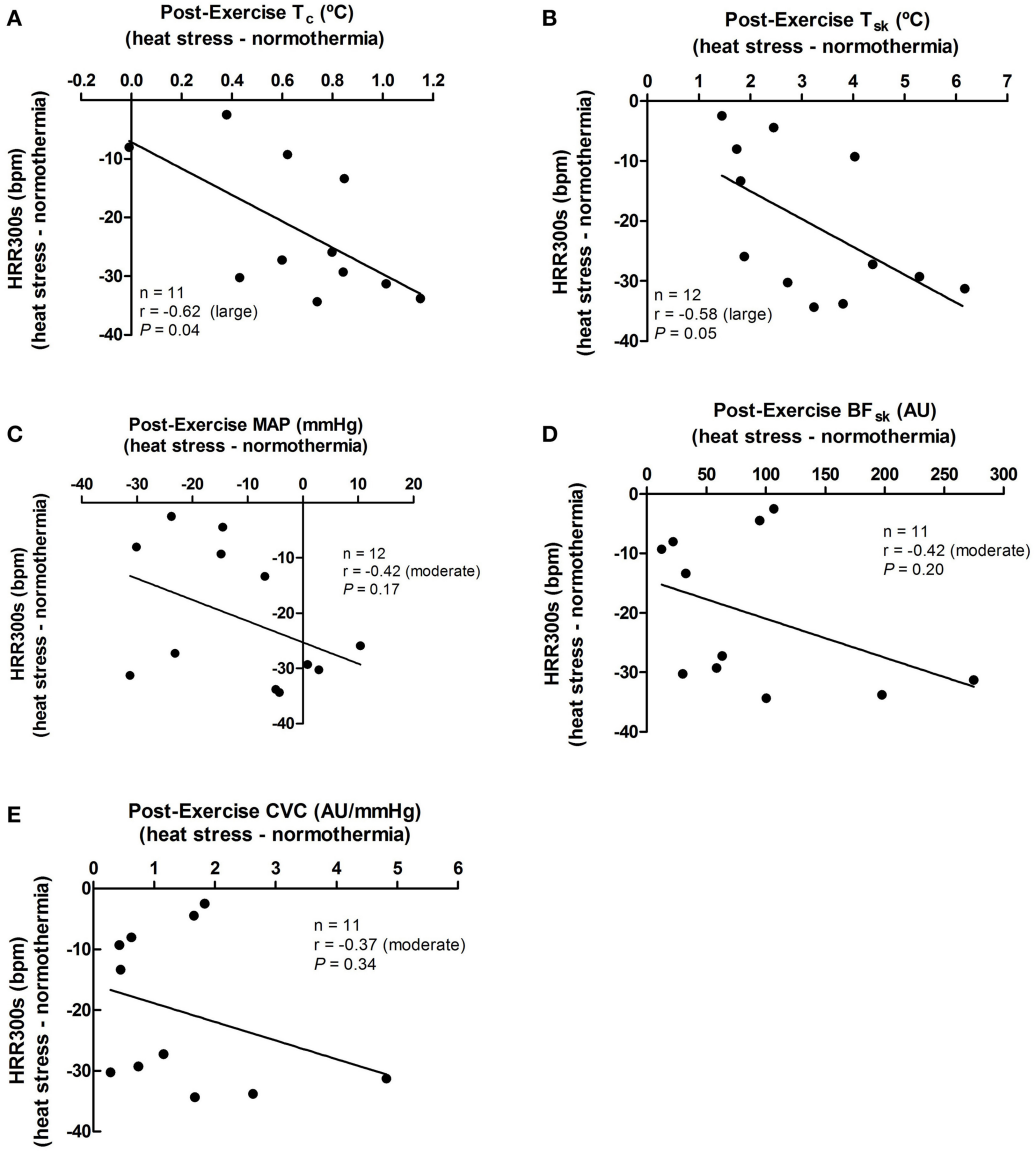
Associations between the effects of heat stress (i.e., heat stress—normothermia) on HRR300s (heart rate decay after 300 s of recovery) and on thermoregulatory and hemodynamic variables. **(A)** T_c_, core temperature; **(B)** T_sk_, skin temperature; **(C)** MAP, mean arterial pressure; **(D)** SKBF, skin blood flow; **(E)** CVC, cutaneous vascular conductance. Due to 1 missing data point of T_c_, SKBF, and CVC, correlations were presented for 11 subjects. Correlations (*r*) were classified as trivial (<0.1), small (0.1–0.3), moderate (>0.3–0.5), large (>0.5–0.7), very large (>0.7–0.9), and extremely large (>0.9).

The effect promoted by heat stress (i.e., HS minus NT) on HRR60s was negatively associated with its effects in post-exercise T_c_ (*P* = 0.02), T_sk_ (*P* = 0.02), SKBF (*P* = 0.01), and CVC (*P* = 0.02), but not with MAP (*P* = 0.69; Figure 4). In addition, the effects of heat stress on HRR300s was negatively associated with its effects on post-exercise T_c_ (*P* = 0.04) and T_sk_ (*P* = 0.05). There were no associations between the effects of heat stress on HRR300s and post-exercise SKBF (*P* = 0.20), MAP (*P* = 0.17), and CVC (*P* = 0.34; Figure 5).

## Discussion

This study assessed the effects of increased thermoregulatory stress, e.g., elevated T_c_, on post-exercise HRR and HRV. The results indicate that heat stress delayed immediate post-exercise HRR and suppressed post-exercise HRV restoration immediately after exercise. In addition, the greater the thermoregulatory stress promoted by HS the greater the delay in post-exercise HRR.

The present findings are in agreement with previous studies showing slower HRR under heating conditions (Leicht et al., 2009; Lynn et al., 2009). Indeed, the performance of exercise and/or recovery under heat stress conditions reduces post-exercise parasympathetic and increases sympathetic modulations of post-exercise HR (Franklin et al., 1993; Lynn et al., 2009). Likewise, strategies to reduce post-exercise T_c_ (e.g., cold water immersion, fanning) have proved to accelerate the return of cardiac autonomic regulation to pre-exercise status (Leicht et al., 2009). However, in these previous studies, either HR (Lynn et al., 2009) or T_c_ (Leicht et al., 2009) were either not matched or were significantly different, respectively, at the end of exercise. In addition, none of the abovementioned studies has focused on the immediate post-exercise recovery period (i.e., first 5 min) that is a very important period for cardiovascular risk prediction and post-exercise cardiac autonomic assessment (Peçanha et al., 2014, 2017a).

In the present study, HS reduced HRR in the fast (i.e., HRR60s and T30) and slow (HRR300s) phases of the immediate post-exercise recovery period (i.e., 0–5 min). In addition, both beat-to-beat (i.e., RMSSD) and overall (i.e., RMS) HRV indices were reduced in HS. This wide effect of heat stress on a number of HRR and HRV indices suggests an important effect of thermoregulation on both parasympathetic reactivation and sympathetic withdrawal after exercise (Peçanha et al., 2014, 2017a). Indeed, the link between thermal stress and parasympathetic and sympathetic responses have been explored in a number of previous studies at rest and during exercise. Crandall et al. (2000) reported a reduction in cardiac parasympathetic modulation during passive heating (increase in T_c_ of ~0.8°C). A dose-response relationship of parasympathetic markers of HRV and acute exposure to progressive thermal stresses (i.e., cool, mild, warm and hot) was demonstrated by Kinugasa and Hirayanagi (1999). With respect to the effects of heating on sympathetic nerve activity, Low et al. (2011) reported increases in muscle and skin sympathetic nerve activity during passive heat stress (increase in T_c_ of ~1.5°C). Similarly, Brenner et al. (1997) observed increased low-to-high frequency ratio of HRV and greater plasma noradrenaline levels during exercise performed under heat stress conditions compared with exercise in thermoneutral conditions (differences in T_c_ of ~ 0.4°C). Thus, the results of the present study extend this relationship between thermoregulation and cardiac autonomic control to immediately post-exercise parasympathetic reactivation and sympathetic withdrawal. Overall, these findings suggest that heat-stress induced reductions in parasympathetic nerve activity and elevations in sympathetic nerve activity restrain the magnitude of parasympathetic reactivation and sympathetic withdrawal after exercise.

Interestingly, there were moderate-to-high correlations between session differences in HRR and in most of the thermoregulatory/hemodynamic responses. Actually, the delay in HRR due to heat stress was correlated with the degree of thermal stress (i.e., increases in T_c_ and T_sk_) and the thermoregulatory-related hemodynamic responses (i.e., increases in SKBF and CVC) produced by heating. These associations, although not necessarily causal, support the hypothesis that the link between thermoregulatory and cardiac autonomic responses after exercise might be secondary to thermoregulatory-mediated hemodynamic changes. In this sense, a thermal-induced redirection of blood flow to cutaneous vessels, as demonstrated by increases in SKBF and CVC, might have reduced central blood volume and ventricular filling during recovery, which could have driven a compensatory sympathetic activation and parasympathetic deactivation (Ryan et al., 2012). Supporting this hypothesis, Cui et al. (2004) have shown exaggerated sympathetic responses to reductions in central blood volume (promoted by lower body negative pressure) under heating conditions in healthy subjects. In addition, the increased HR during recovery (i.e., reduced HRR) in HS might be a reflex response to the reduced MAP during recovery in this session, as suggested by previous investigations (Bennett et al., 1984; Forjaz et al., 2004). However, different from other variables, the effects of HS on MAP and HRR were not correlated. A likely explanation for this absence of association, might reside in the reduced baroreflex sensitivity in the post-exercise period of the HS session, which may have weakened the association between MAP and HR (or HRR) during the post-exercise recovery.

The link between thermoregulation and post-exercise cardiac autonomic recovery might also be direct. Indeed, studies have shown important projections between hypothalamic thermoregulatory areas and cardiovascular control medullary areas (Nakamura et al., 2002; Zaretskaia et al., 2003; Cerri and Morrison, 2005; Morrison and Nakamura, 2011) in the brain. Although speculative, the increase in T_c_ and T_sk_ promoted by the present HS protocol might have stimulated sympathetic and inhibited parasympathetic medullary centers of the central nervous system, culminating with the delayed HRR. Accordingly, Zaretskaia et al. (2003) observed a marked tachycardia after the stimulation of the preoptic hypothalamus in anesthetized rats. The large correlations observed between HRR and temperature (T_c_ and T_sk_) compared to the moderate correlations observed between HRR and hemodynamic (MAP, SKBF, CVC) data, especially for HRR300s, support—at least in part—this direct mechanism. Future studies should further explore these direct and indirect relationships between thermoregulation and cardiovascular neural control, either using experimental models to directly assess/modulate neuronal activity, or employing additional hemodynamic and autonomic assessment in humans, such as central venous pressure, ventricular filling, and direct sympathetic neural recordings. Finally, it is also important to point out that the effect of heat stress on HR regulation might also involve non-autonomic mechanisms related to the direct effect of temperature on sino-atrial node function (Oyston et al., 1989) that was not investigated in the present study.

An important clinical application of immediate HRR is its assumption as an independent predictor of cardiovascular disease and mortality (Cole et al., 1999; Jouven et al., 2005). Jouven et al. (2005) reported increased risk of all cause and cardiovascular mortalities with HRR60s < 25 bpm. Applying this cutoff point in the present results, three subjects changed from non-risk to at risk in the NT and HS sessions, respectively; which highlights the importance of controlling laboratory temperature when evaluating HRR. In addition, the results of the present study shed light on other clinical implications of thermoregulation and HRR. It is well-described that elderly and/or clinical populations (e.g., heart failure patients) present with significantly impaired thermoregulation, leading to increased risks of heat- or cold-related complications (Ogawa et al., 1993; Cui and Sinoway, 2014). Not by chance, these populations are also known to present abnormal cardiovascular responses to exercise and reduced HRR (Peçanha et al., 2014). Georgoulias et al. (2003) reported greater prevalence of abnormal HRR60s in elderly compared with young subjects, and Imai et al. (1994) observed slower HRR30s after exercise in heart failure patients compared with healthy controls. Taken together these information open the perspective that at least part of the reduced HRR observed in clinical populations might involve impaired thermoregulatory responses during or after exercise. If this is true, specific therapies targeting improvement in thermoregulation (e.g., hot/cold baths, exercise in the heat/cold, post-exercise fanning) might benefit HRR in parallel.

A potential practical implication of the effects of increased thermal stress on HRR involves the potential confounding effect of different environmental conditions/severities of thermal stress on HRR when comparing HRR between different groups or over repeated measures across time. For example, daily differences in thermoregulatory responses induced by increase/decrease in temperature and humidity might directly affect HRR. Likewise, seasonal changes in thermoregulation have been reported in mammals (Lovegrove, 2005), and these could potentially affect HRR in the same way. For these reasons, extra caution must be made to ensure very similar environmental conditions and/or thermal stress between sessions/groups in HRR studies.

This study is not without limitations. The results are restricted to healthy young males and future studies should verify the role of thermoregulation on HRR in populations with altered thermoregulatory function (e.g., elderly and populations with chronic diseases). In the present study, the suit was not used in the NT session to avoid undesirable heat storage and maximize thermal differences between NT and HS sessions, however, an effect of wearing the suit cannot be excluded. The exercise protocol involved two different intensities, but this fact might not have affected the results since HR was similar at both intensities between the NT and HS sessions. HRR and HRV are non-invasive measures of cardiac autonomic function, and inferences about parasympathetic and sympathetic functions should be made with caution. Specifically for sympathetic function, the indices employed by the present study are supposed to only partially quantify, at best, this function (Peçanha et al., 2017a). However, doubts remain on the best method to assess cardiovascular sympathetic function, and the best method for resting conditions may not be the most appropriate for immediately post-exercise. In both sessions subjects performed active instead of inactive recovery, and it is possible that interactions between thermoregulation and other mechanisms (e.g., central command and mechanoreflex) might be partly responsible for the observed outcomes (Carter et al., 2002; Shibasaki et al., 2004). That said, it is more than likely that these mechanisms were similar during active recovery in both conditions. The decision to use active recovery was made based on pilot tests due to the risk of significant hypotension during inactive recovery under HS. The use of recumbent ergometers might attenuate the risk of such hypotensive events. Finally, the differences in absolute workloads between NT and HS might explain differences in post-exercise cardiac autonomic recovery. However, greater workloads delay HRR (Imai et al., 1994) and HRR was faster in the NT session which had the higher absolute workload.

In conclusion, heat stress attenuates post-exercise HRR and suppresses post-exercise HRV. These results and the observed associations between HRR and the thermal and hemodynamic impact of heat stress confirm previous models that include thermoregulation as an active mechanism modulating post-exercise cardiac autonomic responses.

## Ethics statement

This study was carried out in accordance with the recommendations of Liverpool John Moores University Research Ethics Committee with written informed consent from all subjects. All subjects gave written informed consent in accordance with the Declaration of Helsinki. The protocol was approved by the Liverpool John Moores University Research Ethics Committee (15/SPS/053).

## Author contributions

TP, CF, and DL participate in the conception and design of the study. TP and DL participate in the acquisition and analysis of the data. TP, CF, and DL were responsible for interpretation of data. TP, CF, and DL contribute to the draft of the paper. TP, CF, and DL critically reviewed the manuscript. All authors approved the final version of the manuscript and agree to be accountable for all aspects of the work.

### Conflict of interest statement

The authors declare that the research was conducted in the absence of any commercial or financial relationships that could be construed as a potential conflict of interest.
